# Sphingosine‐1‐phosphate (S1P) enhances glomerular endothelial cells activation mediated by anti‐myeloperoxidase antibody‐positive IgG

**DOI:** 10.1111/jcmm.13458

**Published:** 2017-11-23

**Authors:** Xiao‐Jing Sun, Min Chen, Ming‐Hui Zhao

**Affiliations:** ^1^ Renal Division Department of Medicine Peking University First Hospital Beijing China; ^2^ Institute of Nephrology Peking University Beijing China; ^3^ Key Laboratory of Renal Disease Ministry of Health of China Beijing China; ^4^ Key Laboratory of Chronic Kidney Disease Prevention and Treatment (Peking University) Ministry of Education Beijing China; ^5^ Peking‐Tsinghua Center for Life Sciences Beijing China

**Keywords:** ANCA, vasculitis, sphingosine‐1‐phosphate

## Abstract

Cumulating evidences suggested an important role of sphingosine‐1‐phosphate (S1P) and its receptors in regulating endothelial barrier integrity. Our previous study revealed that the circulating S1P levels and renal expression of S1PRs correlated with disease activity and renal damage in patients with antineutrophil cytoplasmic antibody (ANCA)‐associated vasculitis (AAV). This study investigated the role of S1P and its receptors in myeloperoxidase (MPO)‐ANCA‐positive IgG‐mediated glomerular endothelial cell (GEnC) activation. The effect of S1P on morphological alteration of GEnCs in the presence of MPO‐ANCA‐positive IgG was observed. Permeability assay was performed to determine endothelial monolayer activation in quantity. Both membrane‐bound and soluble ICAM‐1 and VCAM‐1 levels were measured. Furthermore, antagonists and/or agonists of various S1PRs were employed to determine the role of different S1PRs. S1P enhanced MPO‐ANCA‐positive IgG‐induced disruption of tight junction and disorganization of cytoskeleton in GEnCs. S1P induced further increase in monolayer permeability of GEnC monolayers in the presence of MPO‐ANCA‐positive IgG. S1P enhanced MPO‐ANCA‐positive IgG‐induced membrane‐bound and soluble ICAM‐1/VCAM‐1 up‐regulation of GEnCs. Soluble ICAM‐1 levels in the supernatants of GEnCs stimulated by S1P and MPO‐ANCA‐positive IgG increased upon pre‐incubation of S1PR1 antagonist, while pre‐incubation of GEnCs with the S1PR1 agonist down‐regulated sICAM‐1 level. Blocking S1PR2‐4 reduced sICAM‐1 levels in the supernatants of GEnCs stimulated by S1P and MPO‐ANCA‐positive IgG. Pre‐incubation with S1PR5 agonist could increase sICAM‐1 level in the supernatants of GEnC stimulated by S1P and MPO‐ANCA‐positive IgG. S1P can enhance MPO‐ANCA‐positive IgG‐mediated GEnC activation through S1PR2‐5.

## Introduction

ANCA‐AAV is a group of systemic autoimmune diseases, including microscopic polyangiitis (MPA), granulomatosis with polyangiitis (GPA) and eosinophilic granulomatosis with polyangiitis (EGPA) 1. AAV is characterized by necrotizing inflammation of the small blood vessels, which involves GEnC injury in particular. ANCAs against MPO and proteinase 3 (PR3) are the serological hallmarks of AAV 2, 3. What is noteworthy is that Chinese AAV patients, as demonstrated in our previous studies, are predominantly MPO‐ANCA‐positive 4, 5. Moreover, cumulating evidences reveal that MPO‐ANCAs can induce direct GEnC activation and injury in AAV. Nagao *et al*. 6, 7 reported that MPO‐ANCA could induce up‐regulation of adhesion molecules in GEnCs both *in vivo* and *in vitro*.

S1P is a bioactive sphingolipid and key regulator in vascular inflammation. S1P is the ligand for five G‐protein‐coupled receptors (GPCRs) named S1PR1‐5 8, 9. S1P and its receptors are involved in the pathogenesis of a variety of vascular inflammatory conditions including sepsis, atherosclerosis and ischaemia–reperfusion injury 10, 11, 12, 13, 14. In our previous study, it was found that the levels of circulating S1P were elevated and renal expression of S1PR2‐5 was up‐regulated in AAV patients of active stage, and that the circulating S1P levels and the renal expression of S1PR were associated with renal involvement and disease activity of AAV 15. Moreover, we found that S1P participated in ANCA‐mediated neutrophil activation 16. All these studies indicated a pathogenic role of S1P in AAV 16.

In addition, cumulating evidences suggested an important role of S1P and S1PRs in regulating endothelial barrier integrity in recent years. It was found that different doses of S1P could induce distinct biological effects on endothelial cells and that the final barrier regulating the efficacy of S1P depends on the balance of expression and activation of different S1PRs in endothelial cells. A physiological level of S1P strengthened the barrier integrity of endothelial cells through S1PR1, whereas excessive S1P resulted in disruption of endothelial integrity by activating S1PR2 and S1PR3 17, 18, 19. Given the potential effect of S1P on regulating endothelial barrier function, we hypothesize that S1P might participate in MPO‐ANCA‐positive IgG‐induced GEnC activation through a S1PR‐dependent way. This study aimed to investigate whether S1P contributed to MPO‐ANCA‐positive IgG‐induced GEnC activation, and to explore the roles of different S1PRs in this process.

## Materials and methods

### Cell culture

Primary human GEnC (ScienCell, San Diego, CA, USA) were cultured according to the manufacturer's recommendation. After a confluent endothelial cell monolayer was formed, GEnCs were starved in basal medium supplemented with 1% foetal bovine serum (FBS) for 8 hrs. GEnC in selected wells were then stimulated for 24 hrs with 2 μM S1P (Sigma‐Aldrich, Darmstadt, Germany), which was comparable to the levels of circulating S1P in AAV patients at active stage, as demonstrated by our previous study 15.

### Preparation of IgGs

Normal immunoglobulin (Ig)Gs and MPO‐ANCA‐positive IgGs were prepared from plasma of five healthy volunteers and five active MPO‐ANCA‐positive primary small vessel vasculitis patients, respectively. Plasma was applied to a High‐Trap‐protein G column on an AKTA‐FPLC system (GE Biosciences, South San Francisco, CA, USA) after filtered through syringe filters (Merck Millipore, Darmstadt, Hessen, Germany). Preparation of IgGs was performed according to the methods described previously 20. The endotoxin levels in the IgGs were below the detection limit (0.1 EU/ml) of a commercial ELISA kit (Cambrex Corporation, East Rutherford, NJ, USA). The IgG from each individual patient or healthy volunteer was prepared for subsequent anti‐endothelial cell antibody (AECA) detection and GEnC stimulation. Our research was approved by the clinical research ethics committee of the Peking University First Hospital and in compliance with the Declaration of Helsinki.

### Detection of AECA

The prepared IgGs were further screened for the presence of AECA through an ELISA method described previously 21. In brief, GEnCs were seeded onto 96‐well plates until 90% confluence and then starved of serum. After that, the live GEnCs were incubated with IgG for 24 hrs and then fixed with 4% formaldehyde. After the plates were blocked with 5% bovine serum albumin (BSA), an alkaline phosphatase‐conjugated rabbit anti‐human IgG antibody (Sigma‐Aldrich) was used to detect the bound IgG. P‐Nitrophenyl phosphate (Sigma‐Aldrich) was used for subsequent quantification. Each plate always had a blank control obtained from blocking solution 5% BSA, a standard positive control obtained from an active AAV patient and a negative control obtained from healthy volunteers. Results were expressed as an ELISA ratio (ER). The lower limit for positive AECA binding was defined as ER value greater than the mean + 3S.D. AECA‐positive IgG was excluded with this criterion in our following experiments.

### Measurements of GEnC activation

#### Immunofluorescence staining of zonula occluden‐1 (ZO‐1) and filamentous (F)‐actin

As important makers for endothelial barrier function, the distributions of the tight junction scaffolding protein ZO‐1 and the cytoskeletal F‐actin in GEnCs were observed 22. GEnCs were washed with phosphate‐buffered saline (PBS) and fixed with 4% formaldehyde after relevant treatment. Then GEnCs were permeabilized with 0.5% Triton X‐100, washed in PBS, blocked by 5% BSA and then incubated with a ZO‐1 antibody (Life, Carlsbad, CA, USA) at 4°C overnight. After a thorough wash in PBS, the GEnCs were incubated with a fluorescein isothiocyanate (FITC)‐conjugated secondary antibody (Jackson ImmunoResearch, West Grove, PA, USA) and conjugated rhodamine–phalloidin (Life) for 1 hr. After washing with PBS, the specimens were stained with 4′,6‐diamidino‐2‐phenylindole (DAPI) and eventually mounted with Mowiol. Confocal images were captured by a Zeiss LSM 710 confocal microscope (Zeiss, Jena, Germany) and exported by the ZEN 2012 microscopy software.

#### Permeability assay

We determined the permeability of GEnC monolayers using FITC‐labelled BSA (Sigma‐Aldrich), as described previously 23. We grew GEnCs on the upper chamber of Costar Transwell with 0.5‐μm porous filters until confluent. After relevant stimulation for 24 hrs, we added the tracer protein FITC‐albumin to the upper chamber. After incubated at 37°C for 30 min., samples were collected from both the upper and lower chambers for fluorometric analysis. Dulbecco's phosphate‐buffered saline (D‐PBS) and TNF‐α (2 ng/ml) were used as negative and positive controls, respectively. A microplate fluorescence reader (TristarTM LB941; Berthold, Bad Wildbad, Baden‐Württemberg, Germany) was used to measure fluorescence. Eventually, we use these fluorescence readings to calculate the permeability coefficient, which is indicative of vascular barrier disruption.

#### Measurement of membrane‐bound adhesion molecule expression

Membrane‐bound intercellular adhesion molecule‐1 (mICAM‐1) and membrane‐bound vascular cell adhesion molecule‐1 (mVCAM‐1) expression levels were measured according to the methods described previously 6. GEnC monolayers were pre‐treated exactly as for permeability assay and then fixed with 4% formaldehyde. Fixed GEnCs were pre‐incubated with 5% BSA to block non‐specific binding. Biotinylated sheep anti‐human ICAM‐1 or VCAM‐1 antibodies were then incubated for another hour. Streptavidin‐HRP (R&D, Minneapolis, MN, USA) and then substrate solution were added to detect bound antibodies. Finally, we stopped the reaction by adding sulphuric acid and determined optical density of each well immediately. As for negative controls, the primary antibody was replaced by 5% BSA.

#### Measurement of soluble adhesion molecule in the supernatants

Levels of soluble intercellular adhesion molecule‐1 (sICAM‐1) and soluble vascular cell adhesion molecule‐1 (sVCAM‐1) in the GEnC supernatants were measured using the commercial ELISA kits (R&D). A 96‐well microplate was coated with the diluted ICAM‐1/VCAM‐1 capture antibody. After an overnight incubation and 3 times plate wash, the plate was blocked with 1% BSA to reduce non‐specific binding. Then we added sample or standards to the plate. After 2 hrs incubation at room temperature and three times wash, ICAM‐1/VCAM‐1detection antibody was added to each well. After another 2 hrs incubation and plate wash, streptavidin–horseradish peroxidase (HRP) was added to each well. After another incubation, substrate solution was added. Then the reaction was stopped by adding sulphuric acid. Eventually, the optical density of each well was determined immediately using a microplate reader set to 570 nm.

### Expression of S1P receptors in human GEnCs

Reverse transcription‐polymerase chain reaction (RT‐PCR) was performed to detect S1PRs expression in GEnCs. Total RNA was extracted according to the manufacturer's instructions (Thermo, Waltham, MA, USA), which was subjected to reverse‐transcribed using a commercial reverse transcription system (Promega, Wisconsin, WI, USA). Quantitative real‐time PCR was then performed with ABI Prism 7500 sequence detecting system (Applied Biosystems, Foster City, CA, USA), and the PCR products were size‐fractionated in a 2% agarose gel. The primers for PCR amplifications were as follows: S1PR1 (Forward: 5′‐CACTCTGACCAACAAGGAGATG‐3′, Reverse: 5′‐GATGATGGGTCGCTTGAATTTG‐3′); S1PR2 (Forward: 5′‐AAGTTCCACTCGGCAATGTA‐3′, Reverse: 5′‐AGCCAGAGAGCAAGGTATTG‐3′); S1PR3 (Forward: 5′‐TCTCCGAAGGTCAAGGAAGA‐3′, Reverse: 5′‐TCAGTTGCAGAAGATCCCATTC‐3′); S1PR4 (Forward: 5′‐CTGAA‐GACGGTGCTGATGAT‐3, Reverse: 5′‐CAGAGGTTGGAGCCAAAGA‐3′); S1PR5 (Forward: 5′‐GGTCATCGTCCTGCATTACA‐3′, Reverse: 5′‐CTAGATTCTCTAGCACGATGAAGG‐3′); and β‐actin (Forward: 5′‐GGACCT‐GACTGACTACCTCAT‐3′, Reverse: 5′‐CGTAGCACAGCTTCTCCTTAAT‐3′).

### Inhibition of S1P receptors

SEW2871 (SEW; Tocris, Louis, MO, USA) is a novel, potent and selective S1PR1 agonist which activates S1PR1, but does not activate S1PR2‐5 at concentrations even up to 10 μM 24. W146 (Tocris) is a highly specific S1PR1 antagonist which displays no effect at S1PR2, S1PR3 or S1PR5 25, 26. JTE013 (JTE; Tocris) is a highly selective S1PR2 antagonist. Even at concentrations up to 10 μM, JTE013 displays only 4.2% inhibition of S1PR3 and does not antagonize S1PR1 27, 28. TY52156 (TY; Tocris) shows submicromolar potency and a high degree of selectivity for S1P3 receptor 29, 30. CYM50358 (CYM; Tocris) is a potent S1PR4 antagonist which displays selectivity for S1PR4 against S1PR1, S1PR2, S1PR3 and S1PR5 31. A971432 is a potent and selective S1PR5 agonist which exhibits 60‐fold selectivity over S1PR1, and over 1600‐fold selectivity over S1PR2‐4 32.

In S1P‐induced sICAM‐1 expression assay, GEnCs were incubated with the aforementioned S1PR agonists or antagonists for different doses and time‐points. Eventually 0.1 μM of S1PR agonists or antagonists at 15 min. was selected for the experiments because of the highest inhibition/increase rate (Fig. S1‐S2). In addition, it has been validated that these S1PR antagonists or agonists are selective for their respective receptors at the 0.1 μM concentration according to the previous studies 25, 26, 27, 28, 29, 30, 31, 32.

### Statistical analysis

Kurtosis and skewness were used to evaluate the normality of the data (both the absolute values were <3). Differences in quantitative parameters between groups were analysed using the *t*‐test, and mean ± S.D. were presented as descriptive statistics for normally distributed data. Differences in quantitative parameters between groups were analysed using the nonparametric test, and median and interquartile range (IQR) were presented as descriptive statistics for non‐normally distributed data. For normally distributed data, differences in quantitative parameters between groups were assessed with one‐way anova, and for non‐normally distributed data, differences in quantitative parameters between groups were assessed with Kruskal–Wallis test. If *P *<* *0.05, differences were considered statistically significant. Data analysis was performed with SPSS version 13.0 (SPSS Inc., Chicago, IL, USA).

## Results

### S1P induces morphological alteration in GEnC monolayers

**Figure 1:**
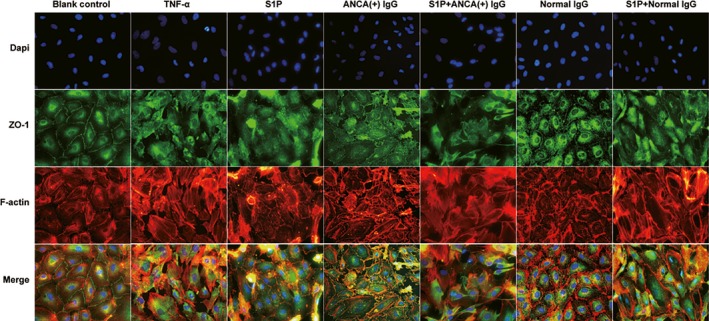
S1P could induce alterations in cellular morphology of GEnCs in the presence of MPO‐ANCA‐positive IgG.

Double immunofluorescence staining of ZO‐1 and F‐actin was performed to observe the structure of the tight junction and cytoskeleton in GEnCs. The results revealed that the application of S1P or MPO‐ANCA‐positive IgG alone could induce the disruption of tight junction structure and the improper distribution of F‐actin compared with untreated cells. Moreover, combined application of S1P and MPO‐ANCA‐positive IgG induced further disruption of tight junction and disorganization of cytoskeleton compared with all the above‐mentioned cell groups (Fig. 1). These data suggested that S1P, with pathophysiological concentration of active AAV patients, could enhance MPO‐ANCA‐positive IgG‐induced endothelial activation.

### S1P induces increased endothelial permeability of GEnC monolayers

**Figure 2:**
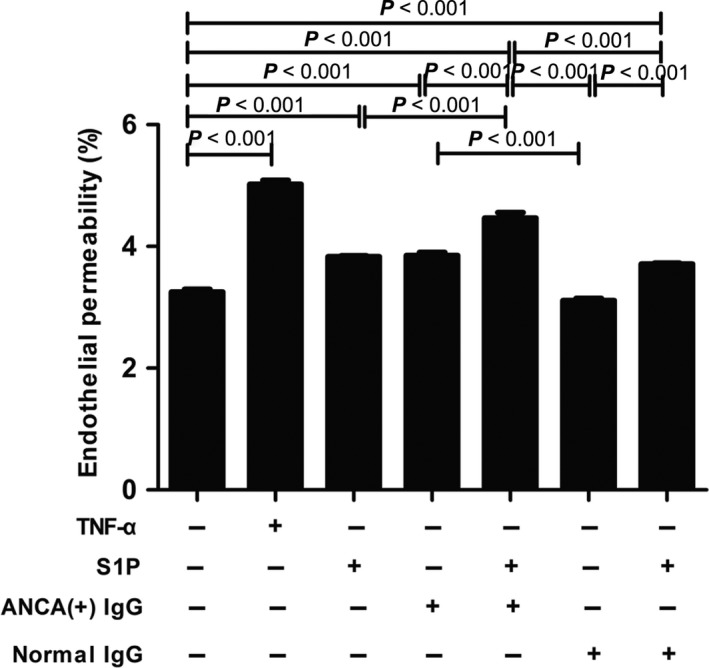
S1P could induce increased endothelial permeability of GEnC monolayers in the presence of MPO‐ANCA‐positive IgG. Bars represent mean ± S.D. of repeated measurements of five independent experiments or donors.

To determine the effect of S1P on monolayer permeability in GEnCs, a transwell system and a FITC‐labelled BSA were used. The results demonstrated that compared with untreated cells, monolayer permeability increased significantly in GEnCs stimulated by S1P and MPO‐ANCA‐positive IgG (4.47 ± 0.20% versus 3.25 ± 0.10%, *P *<* *0.001). Monolayer permeability also increased in GEnCs stimulated by S1P or MPO‐ANCA‐positive IgG alone (3.83 ± 0.04% versus 3.25 ± 0.10%, *P *<* *0.001; 3.85 ± 0.12% versus 3.25 ± 0.10%, *P *<* *0.001, respectively). Moreover, compared with the above cells, monolayer permeability still increased significantly in GEnCs stimulated by S1P and MPO‐ANCA‐positive IgG (4.47 ± 0.20% versus 3.25 ± 0.10%, *P *<* *0.001; 4.47 ± 0.20% versus 3.83 ± 0.04%, *P *<* *0.001; 4.47 ± 0.20% versus 3.85 ± 0.12%, *P *<* *0.001, respectively) (Fig. 2). These data suggested that S1P enhanced MPO‐ANCA‐positive IgG‐mediated increasing of GEnC permeability.

### S1P up‐regulates membrane‐bound adhesion molecule expression on GEnC

**Figure 3:**
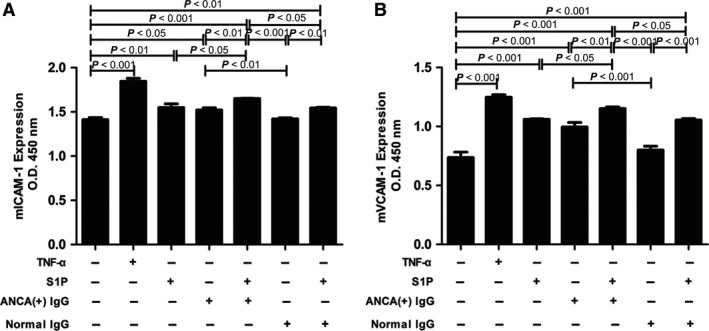
S1P could up‐regulate membrane‐bound adhesion molecule expression levels of GEnC in the presence of MPO‐ANCA‐positive IgG. **A**. S1P could up‐regulate mICAM‐1 expression on GEnC in the presence of MPO‐ANCA‐positive IgG. **B**. S1P could up‐regulate mVCAM‐1 expression on GEnC in the presence of MPO‐ANCA‐positive IgG. Bars represent mean ± S.D. of repeated measurements of five independent experiments or donors.

Adhesion molecules such as ICAM‐1 and VCAM‐1 play an important role in neutrophil adhesion to endothelium, which is essential in the pathogenesis of AAV 22. We detected mICAM‐1 and mVCAM‐1 expression levels using a cell ELISA assay, and we found that compared with untreated cells, cells stimulated by S1P or MPO‐ANCA‐positive IgG alone, the mICAM‐1 levels increased significantly in GEnCs stimulated by S1P and MPO‐ANCA‐positive IgG (1.65 ± 0.01 versus 1.41 ± .053, *P *<* *0.001; 1.65 ± 0.01 versus 1.55 ± 0.09, *P *<* *0.05; 1.65 ± 0.01 versus 1.52 ± 0.05, *P *<* *0.01, respectively. Data was shown as OD values). The levels of mVCAM‐1 also increased significantly in GEnCs stimulated by S1P and MPO‐ANCA‐positive IgG compared with unstimulated cells, cells stimulated by S1P or MPO‐ANCA‐positive IgG alone (1.15 ± 0.03 versus 0.74 ± 0.10, *P *<* *0.001; 1.15 ± 0.03 versus 1.06 ± .013, *P *<* *0.05; 1.15 ± 0.03 versus 1.00 ± 0.09, *P *<* *0.01, respectively. Data were shown as OD values) (Fig. 3). Collectively, these data illustrate that S1P enhanced MPO‐ANCA‐positive IgG‐mediated mICAM‐1 and mVCAM‐1 up‐regulation on GEnCs.

### S1P increases soluble adhesion molecule level in the supernatants

**Figure 4:**
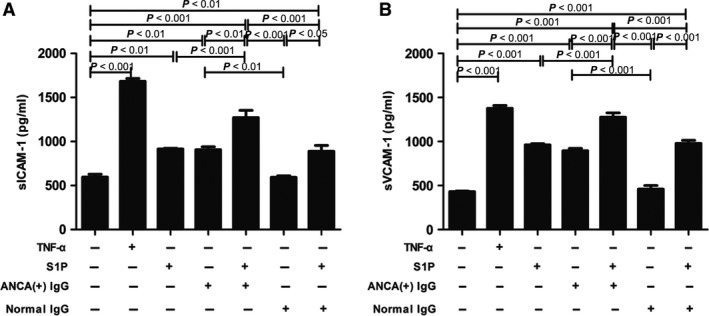
S1P could up‐regulate soluble adhesion molecule expression levels in the supernatants of GEnC in the presence of MPO‐ANCA‐positive IgG. **A**. S1P could up‐regulate sICAM‐1 level in the supernatants of GEnC in the presence of MPO‐ANCA‐positive IgG. **B**. S1P could up‐regulate sVCAM‐1 level in the supernatants of GEnC in the presence of MPO‐ANCA‐positive IgG. Bars represent mean ± S.D. of repeated measurements of five independent experiments or donors.

Soluble adhesion molecules such as sICAM‐1 and sVCAM‐1 levels in the supernatants were measured with commercial ELISA kits. It was found that compared with unstimulated cells, cells stimulated by S1P or MPO‐ANCA‐positive IgG alone, the levels of sICAM‐1 increased significantly in GEnCs treated with S1P and MPO‐ANCA‐positive IgG (1271.93 ± 185.33 pg/ml versus 597.02 ± 72.86 pg/ml, *P *<* *0.001; 1271.93 ± 185.33 pg/ml versus 915.89 ± 20.22 pg/ml, *P *<* *0.001; 1271.93 ± 185.33 pg/ml versus 907.61 ± 73.18 pg/ml, *P *<* *0.01, respectively). The levels of sVCAM‐1 also increased significantly in GEnCs stimulated by S1P and MPO‐ANCA‐positive IgG compared with unstimulated cells, cells stimulated by S1P or MPO‐ANCA‐positive IgG alone (1276.77 ± 109.68 pg/ml versus 432.65 ± 13.33 pg/ml, *P *<* *0.001; 1276.77 ± 109.68 pg/ml versus 961.92 ± 32.85 pg/ml, *P *<* *0.001; 1276.77 ± 109.68 pg/ml versus 896.66 ± 57.13 pg/ml, *P *<* *0.001, respectively) (Fig. 4). Collectively, these data reveal that S1P enhanced MPO‐ANCA‐positive IgG‐induced sICAM‐1 and sVCAM‐1 up‐regulation in the GEnC supernatants.

### Differential activation of S1PRs mediates the S1P‐induced endothelial activation

First, RT‐PCR was performed to confirm the expression of S1PRs in GEnCs, and we found that GEnCs express all the five types of S1P receptors, that is S1PR1‐5 (Fig. S4). To further determine the role of S1PRs through which S1P exert its effect, GEnCs were pre‐incubated with various S1PR antagonists or agonists for 15 min. before stimulation with S1P and MPO‐ANCA‐positive IgG, and the sICAM‐1 level in the supernatants was measured. Pre‐incubation of GEnCs with the S1PR1 antagonist W146 significantly increased sICAM‐1 levels in the supernatants of GEnCs stimulated by S1P and MPO‐ANCA‐positive IgG. The sICAM‐1 levels in the supernatants of GEnCs stimulated by S1P and MPO‐ANCA‐positive IgG were 1228.88 ± 118.63 pg/ml, which increased to 1427.92 ± 81.58 pg/ml upon pre‐incubation with S1PR1 antagonist W146 (compared with that without the antagonist, *P *<* *0.01, with the increase rate of 16.20 ± 6.64%). On the contrary, pre‐incubation of GEnCs with the S1PR1 agonist SEW significantly decreased sICAM‐1 level in the supernatants of GEnCs stimulated by S1P and MPO‐ANCA‐positive IgG, with the inhibition rate of 13.02 ± 7.93%.

Pre‐treatment with S1PR2 antagonist JTE significantly reduced sICAM‐1 level in the supernatants of GEnCs stimulated by S1P and MPO‐ANCA‐positive IgG, with the inhibition rate of 24.04 ± 6.55%.

The sICAM‐1 levels reduced from 1368.44 ± 277.54 pg/ml in the supernatants of GEnCs stimulated by S1P and MPO‐ANCA‐positive IgG to 1006.27 ± 83.09 pg/ml, upon pre‐incubation with S1PR3 antagonist TY, with the inhibition rate of 26.47 ± 6.07%.

Pre‐treatment with S1PR4 antagonist CYM significantly decreased sICAM‐1 level in the supernatants of GEnCs stimulated by S1P and MPO‐ANCA‐positive IgG, with the inhibition rate of 21.96 ± 5.60%.

**Figure 5:**
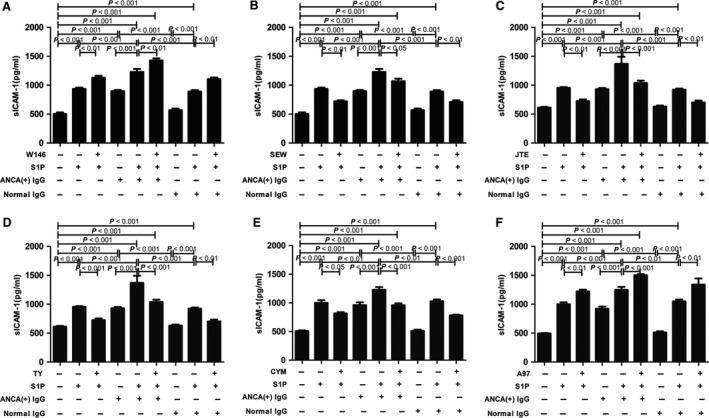
Differential activation of S1PRs mediated the S1P‐induced sICAM‐1 up‐regulation of GEnCs in the presence of MPO‐ANCA‐positive IgG. **A**. S1PR1 antagonist W146 significantly increased sICAM‐1 level in the supernatants of GEnC stimulated by S1P plus MPO‐ANCA‐positive IgG. **B**. S1PR1 agonist SEW significantly reduced sICAM‐1 level in the supernatants of GEnC stimulated by S1P plus MPO‐ANCA‐positive IgG. **C**. S1PR2 antagonist JTE significantly reduced sICAM‐1 level in the supernatants of GEnC stimulated by S1P plus MPO‐ANCA‐positive IgG. **D**. S1PR3 antagonist TY significantly reduced sICAM‐1 level in the supernatants of GEnC stimulated by S1P plus MPO‐ANCA‐positive IgG. **E**. S1PR4 antagonist CYM significantly reduced sICAM‐1 level in the supernatants of GEnC stimulated by S1P plus MPO‐ANCA‐positive IgG. **F**. S1PR5 agonist A97 significantly increased sICAM‐1 level in the supernatants of GEnC stimulated by S1P plus MPO‐ANCA‐positive IgG. Bars represent mean ± S.D. of repeated measurements of five independent experiments or donors.

For S1PR5 agonist, sICAM‐1 level in the supernatants was 1249.18 ± 111.50 pg/ml in the supernatants of GEnCs stimulated by S1P and MPO‐ANCA‐positive IgG, which increased to 1502.25 ± 40.11 pg/ml upon pre‐incubation with S1PR5 agonist A97, with the increase rate of 20.26 ± 3.21% (Fig. 5, Fig. S3 ).

Collectively, these data revealed that S1P enhanced MPO‐ANCA‐positive IgG‐mediated sICAM‐1 up‐regulation of GEnCs through S1PR2–5, whereas S1PR1 induced sICAM‐1 down‐regulation of GEnCs stimulated by S1P and MPO‐ANCA‐positive IgG. Differential activation of S1PRs mediated the S1P‐induced endothelial activation in the presence of MPO‐ANCA‐positive IgG.

## Discussion

In this study, we observed that S1P could enhance MPO‐ANCA‐positive IgG‐induced GEnC activation. Furthermore, we found that activation of S1PR2–5 was implicated in the S1P‐induced endothelial activation in the presence of MPO‐ANCA‐positive IgG.

According to the study by Nagao *et al*., anti‐MPO antibody activates endothelial cells *via* moesin. Moesin, with the full name of membrane‐organizing extension spike protein, shares certain similar sequences with those on the N‐terminal region of the MPO heavy chain 7. Furthermore, MPO‐ANCA from patients with active AAV had high reactivity to the aforementioned sequences in human 33. Although MPO is not expressed in endothelial cells, anti‐MPO antibody could activate GEnCs by recognizing moesin 34. In our previous study, we also confirmed the binding of anti‐MPO antibody to moesin in human GEnCs using various methods. Furthermore, binding of anti‐MPO antibody to moesin was confirmed to up‐regulate adhesion molecules and to induce hyperpermeability of human GEnCs 35.

Recently, a number of studies have documented that alterations in intercellular junctional integrity and cytoskeletal organization interact can regulate endothelial barrier homeostasis dynamically. Disruption of tight junction leads to gap formation and increased endothelial permeability, whereas assembly of tight junction enhances barrier integrity. Therefore, assembly of tight junction which consists of a complex of proteins such as occludin, claudin and junctional adhesion molecule (JAM) is essential for the maintenance of endothelial barrier function 36, 37, 38, 39. In our current study, we found that S1P induced disruption of ZO‐1‐indicated tight junction and disorganization of phalloidin‐indicated cytoskeleton, therefore increasing glomerular endothelial permeability in the presence of MPO‐ANCA‐positive IgG. However, the key proteins of tight junction involved in this process and the underlying mechanism require further investigation.

In this study, we also found that S1P contributed to the up‐regulation of both membrane‐bound and soluble ICAM‐1/VCAM‐1 expression levels of GEnCs in the presence of MPO‐ANCA‐positive IgG. ICAM‐1 and VCAM‐1, not only are biomarkers for endothelial cell activation and injury 40, but also might promote neutrophil adhesion and damage to endothelial cells. Evidences have revealed that neutrophils adhere only in response to the local expression of adhesion molecules on the endothelial surface and release of cytokines along the basal side of endothelium 41, 42. Therefore, we speculate that in the presence of MPO‐ANCA‐positive IgG, S1P‐induced ICAM‐1/VCAM‐1 up‐regulation may promote neutrophil adhesion to GEnCs, which may aggravate the endothelial barrier dysfunction.

How MPO‐ANCA and S1P interact to cause GEnCs activation in the *in vitro* setting is not fully clear yet. Moesin, which can be recognized by MPO‐ANCA in GEnCs, is previously described as a cytoskeletal protein that belongs to the ezrin–radixin–moesin (ERM) family 43. Recently, it was reported that S1P could cause acute and potent ERM activation. S1P was confirmed to activate moesin at nanomolar concentrations within a few minutes of treatment. Multiple protein kinase C (PKC) isoforms were shown to be responsible for moesin phosphorylation in response to S1P 44, 45, 46. Therefore, we speculate that moesin recognized by MPO‐ANCA could be further activated by S1P, which might cause enhanced GEnC activation *in vitro*.

All the five types of S1P receptors, S1PR1‐5, have high affinity for S1P 8. Recent studies revealed that the balance of the expression and activation of different S1PRs in endothelial cells plays a key role in regulating endothelial integrity. Physiological concentrations of S1P preserve endothelial barrier function by activating S1PR1, whereas excessive S1P induced endothelial malfunction by activating S1PR2 17. S1PR3 promotes leucocyte rolling, while leucocyte rolling is enhanced in endothelial‐specific S1PR1 knockout mice 47. In the present study, it was found that under pathophysiological concentration of S1P in active AAV patients, the activation of S1PR2–5 dominates the S1P‐induced MPO‐ANCA‐positive IgG‐mediated endothelial activation, whereas S1PR1 exerts opposite effect during this process. It indicated that the imbalance between different S1PR activation might participate in the development of AAV.

Regarding the role of S1PRs in regulating vascular integrity, most studies concentrated on S1PR1‐3, while the effect of S1PR4 and S1PR5 on endothelial barrier function is far from clear. Our previous study found that compared with normal controls, the expression levels of S1PR4 and S1PR5 in AAV patients in glomeruli were up‐regulated. Moreover, correlation analysis revealed that the S1PR4 expression level correlated with the proportion of cellular crescents in renal specimens from AAV patients 15. In this current study, we demonstrated that activation of S1PR4 and S1PR5 participated in S1P‐induced MPO‐ANCA‐positive IgG‐mediated endothelial activation, which extends our knowledge about the function of S1PR4 and S1PR5.

Recent studies demonstrated that the S1PRs can regulate small GTPases of the Rho family differentially, especially Rho and Rac, which are essential in cytoskeletal rearrangement, therefore participate in maintenance/disruption of cell barrier integrity 39, 48. Activation of S1PR1 promotes cytoskeletal rearrangement in a Rac GTPase‐dependent manner, which is necessary for S1P‐induced endothelial barrier enhancement. On the contrary, blockade of S1PR2 or S1PR3 significantly inhibited RhoA activation, therefore alleviating endothelial barrier disruption 19, 49, 50. Whether similar mechanisms exist in AAV remains further exploration.

## Conclusions

In conclusion, S1P was found to be able to enhance MPO‐ANCA‐positive IgG‐mediated GEnC activation through S1PR2‐5. The current findings are of help to figure out the pathogenic role of S1P in AAV, thus providing potential clues for intervention strategies.

## Conflict of interest

There is no conflict of interest to declare.

## Supporting information


**Figure S1.** Dose effect of S1P on sICAM‐1 expression in the supernatants of GEnCs.

**Figure S2.** Dose effect of S1PR agonists or antagonists on S1P‐induced sICAM‐1 expression.

**Figure S3.** Effect of S1PR agonists on sICAM‐1 level in the supernatants of GEnC stimulated by S1P plus MPO‐ANCA‐positive IgG.

**Figure S4.** Expression of S1PR1–5 in GEnCs were measured by RT‐PCR.

## References

[1] Jennette JC , Falk RJ , Bacon PA , *et al* Revised International Chapel Hill consensus conference Nomenclature of Vasculitis. Arthritis Rheum. 2013; 65: 1–11. 23045170 10.1002/art.37715

[2] Segelmark M , Wieslander J . IgG subclasses of antineutrophil cytoplasm autoantibodies (ANCA). Nephrol Dial Transplant. 1993; 8: 696–702. 8414154 10.1093/ndt/8.8.696

[3] Falk RJ , Terrell RS , Charles LA , *et al* Anti‐neutrophil cytoplasmic autoantibodies induce neutrophils to degranulate and produce oxygen radicals *in vitro* . Proc Natl Acad Sci USA. 1990; 87: 4115–9. 2161532 10.1073/pnas.87.11.4115 PMC54058

[4] Chen M , Yu F , Zhang Y , *et al* Characteristics of Chinese patients with Wegener's granulomatosis with anti‐myeloperoxidase autoantibodies. Kidney Int. 2005; 68: 2225–9. 16221222 10.1111/j.1523-1755.2005.00679.x

[5] Li ZY , Chang DY , Zhao MH , *et al* Predictors of treatment resistance and relapse in antineutrophil cytoplasmic antibody‐associated vasculitis: a study of 439 cases in a single Chinese center. Arthritis Rheumatol. 2014; 66: 1920–6. 24623469 10.1002/art.38621

[6] Nagao T , Matsumura M , Mabuchi A , *et al* Up‐regulation of adhesion molecule expression in glomerular endothelial cells by anti‐myeloperoxidase antibody. Nephrol Dial Transplant. 2007; 22: 77–87. 17005520 10.1093/ndt/gfl555

[7] Nagao T , Suzuki K , Utsunomiya K , *et al* Direct activation of glomerular endothelial cells by anti‐moesin activity of anti‐myeloperoxidase antibody. Nephrol Dial Transplant. 2011; 26: 2752–60. 21378392 10.1093/ndt/gfr032

[8] Proia RL , Hla T . Emerging biology of sphingosine‐1‐phosphate: its role in pathogenesis and therapy. J Clin Invest. 2015; 125: 1379–87. 25831442 10.1172/JCI76369 PMC4409021

[9] Jo SK , Bajwa A , Awad AS , *et al* Sphingosine‐1‐phosphate receptors: biology and therapeutic potential in kidney disease. Kidney Int. 2008; 73: 1220–30. 18322542 10.1038/ki.2008.34 PMC2614447

[10] Winkler MS , Nierhaus A , Poppe A , *et al* Sphingosine‐1‐phosphate (S1P): a potential biomarker and therapeutic target for endothelial dysfunction and sepsis? Shock. 2017; 47: 666–72. 27922551 10.1097/SHK.0000000000000814

[11] Winkler MS , Nierhaus A , Holzmann M , *et al* Decreased serum concentrations of sphingosine‐1‐phosphate in sepsis. Crit Care. 2015; 19: 372. 26498205 10.1186/s13054-015-1089-0 PMC4620595

[12] Stone ML , Sharma AK , Zhao Y , *et al* Sphingosine‐1‐phosphate receptor 1 agonism attenuates lung ischemia‐reperfusion injury. Am J Physiol Lung Cell Mol Physiol. 2015; 308: L1245–52. 25910934 10.1152/ajplung.00302.2014 PMC4587601

[13] Potì F , Ceglarek U , Burkhardt R , *et al* SKI‐II–a sphingosine kinase 1 inhibitor–exacerbates atherosclerosis in low‐density lipoprotein receptor‐deficient (LDL‐R‐/‐) mice on high cholesterol diet. Atherosclerosis. 2015; 240: 212–5. 25801013 10.1016/j.atherosclerosis.2015.03.020

[14] Zhang G , Yang L , Kim GS , *et al* Critical role of sphingosine‐1‐phosphate receptor 2 (S1PR2) in acute vascular inflammation. Blood. 2013; 122: 443–55. 23723450 10.1182/blood-2012-11-467191 PMC3716205

[15] Sun XJ , Wang C , Zhang LX , *et al* Sphingosine‐1‐phosphate and its receptors in anti‐neutrophil cytoplasmic antibody–associated vasculitis. Nephrol Dial Transplant. 2017; 32: 1313–22. 28206609 10.1093/ndt/gfw427

[16] Hao J , Huang YM , Zhao MH , *et al* The interaction between C5a and sphingosine‐1‐phosphate in neutrophils for antineutrophil cytoplasmic antibody mediated activation. Arthritis Res Ther. 2014; 16: R142. 25000985 10.1186/ar4604 PMC4227110

[17] Li Q , Chen B , Zeng C , *et al* Differential activation of receptors and signal pathways upon stimulation by different doses of sphingosine‐1‐phosphate in endothelial cells. Exp Physiol. 2015; 100: 95–107. 25557733 10.1113/expphysiol.2014.082149

[18] Christoffersen C , Obinata H , Kumaraswamy SB , *et al* Endothelium‐protective sphingosine‐1‐phosphate provided by HDL‐associated apolipoprotein M. Proc Natl Acad Sci USA. 2011; 108: 9613–8. 21606363 10.1073/pnas.1103187108 PMC3111292

[19] Skoura A , Hla T . Regulation of vascular physiology and pathology by the S1P2 receptor subtype. Cardiovasc Res. 2009; 82: 221–8. 19287048 10.1093/cvr/cvp088 PMC2721650

[20] Schreiber A , Rolle S , Peripelittchenko L , *et al* Phosphoinositol 3‐kinase‐gamma mediates antineutrophil cytoplasmic autoantibody‐induced glomerulonephritis. Kidney Int. 2010; 77: 118–28. 19907415 10.1038/ki.2009.420

[21] Carvalho D , Savage CO , Black CM , *et al* IgG antiendothelial cell autoantibodies from scleroderma patients induce leukocyte adhesion to human vascular endothelial cells *in vitro* . J Clin Invest. 1996; 97: 111–9. 8550821 10.1172/JCI118377 PMC507068

[22] Hartsock A , Nelson WJ . Adherens and tight junctions: structure, function and connections to the actin cytoskeleton. Biochim Biophys Acta. 2008; 1778: 660–9. 17854762 10.1016/j.bbamem.2007.07.012 PMC2682436

[23] Tinsley JH , Wu MH , Ma W , *et al* Activated neutrophils induce hyperpermeability and phosphorylation of adherens junction proteins in coronary venular endothelial cells. J Biol Chem. 1999; 274: 24930–4. 10455168 10.1074/jbc.274.35.24930

[24] Hale JJ , Lynch CL , Neway W , *et al* A rational utilization of high‐throughput screening affords selective, orally bioavailable 1‐benzyl‐3‐carboxyazetidine sphingosine‐1‐phosphate‐1 receptor agonists. J Med Chem. 2004; 47: 6662–5. 15615513 10.1021/jm0492507

[25] Sanna MG , Wang SK , Gonzalez‐Cabrera PJ , *et al* Enhancement of capillary leakage and restoration of lymphocyte egress by a chiral S1P1 antagonist *in vivo* . Nat Chem Biol. 2006; 2: 434–41. 16829954 10.1038/nchembio804

[26] Sanna MG , Liao J , Jo E , *et al* Sphingosine 1‐phosphate (S1P) receptor subtypes S1P1 and S1P3, respectively, regulate lymphocyte recirculation and heart rate. J Biol Chem. 2004; 279: 13839–48. 14732717 10.1074/jbc.M311743200

[27] Inoki I , Takuwa N , Sugimoto N , *et al* Negative regulation of endothelial morphogenesis and angiogenesis by S1P2 receptor. Biochem Biophys Res Commun. 2006; 346: 293–300. 16756949 10.1016/j.bbrc.2006.05.119

[28] Parrill AL , Sardar VM , Yuan H , *et al* Sphingosine 1‐phosphate and lysophosphatidic acid receptors: agonist and antagonist binding and progress toward development of receptor‐specific ligands. Semin Cell Dev Biol. 2004; 15: 467–76. 15271292 10.1016/j.semcdb.2004.05.006

[29] Hirata N , Yamada S , Shoda T , *et al* Sphingosine‐1‐phosphate promotes expansion of cancer stem cells *via* S1PR3 by a ligand‐independent Notch activation. Nat Commun. 2014; 5: 4806. 25254944 10.1038/ncomms5806

[30] Murakami A , Takasugi H , Ohnuma S , *et al* Sphingosine 1‐phosphate (S1P) regulates vascular contraction *via* S1P3 receptor: investigation based on a new S1P3 receptor antagonist. Mol Pharmacol. 2010; 77: 704–13. 20097776 10.1124/mol.109.061481

[31] Guerrero M , Urbano M , Velaparthi S , *et al* Discovery, design and synthesis of the first reported potent and selective sphingosine‐1‐phosphate 4 (S1P4) receptor antagonists. Bioorg Med Chem Lett. 2011; 21: 3632–6. 21570287 10.1016/j.bmcl.2011.04.097 PMC3107912

[32] Hobson AD , Harris CM , van der Kam EL , *et al* Discovery of A‐971432, An Orally Bioavailable Selective Sphingosine‐1‐Phosphate Receptor 5 (S1P5) Agonist for the Potential Treatment of Neurodegenerative Disorders. J Med Chem. 2015; 58: 9154–70. 26509640 10.1021/acs.jmedchem.5b00928

[33] Suzuki K , Kobayashi S , Yamazaki K , *et al* Analysis of risk epitopes of anti‐neutrophil antibody MPO‐ANCA in vasculitis in Japanese population. Microbiol Immunol. 2007; 51: 1215–20. 18094540 10.1111/j.1348-0421.2007.tb04017.x

[34] Pendergraft WF , Alcorta DA , Segelmark M , *et al* ANCA antigens, proteinase 3 and myeloperoxidase, are not expressed in endothelial cells. Kidney Int. 2000; 57: 1981–90. 10792617 10.1046/j.1523-1755.2000.00048.x

[35] Deng H , Wang C , Chang DY , *et al* High mobility group box‐1 contributes to anti‐myeloperoxidase antibody‐induced glomerular endothelial cell injury through a moesin‐dependent route. Arthritis Res Ther. 2017; 19: 125. 28587670 10.1186/s13075-017-1339-4 PMC5461689

[36] Wittchen ES , Haskins J , Stevenson BR . Protein interactions at the tight junction. Actin has multiple binding partners, and ZO‐1 forms independent complexes with ZO‐2 and ZO‐3. J Biol Chem. 1999; 274: 35179–85. 10575001 10.1074/jbc.274.49.35179

[37] Günzel D , Yu AS . Claudins and the modulation of tight junction permeability. Physiol Rev. 2013; 93: 525–69. 23589827 10.1152/physrev.00019.2012 PMC3768107

[38] Nyqvist D , Giampietro C , Dejana E . Deciphering the functional role of endothelial junctions by using *in vivo* models. EMBO Rep. 2008; 9: 742–7. 18600233 10.1038/embor.2008.123 PMC2515211

[39] Radeva MY , Waschke J . Mind the gap: mechanisms regulating the endothelial barrier. Acta Physiol (Oxf). 2017; [Epub ahead of print] https://doi.org/10.1111/apha.12860. 10.1111/apha.12860 28231640

[40] Page AV , Liles WC . Biomarkers of endothelial activation/dysfunction in infectious diseases. Virulence. 2013; 4: 507–16. 23669075 10.4161/viru.24530 PMC5359744

[41] Halbwachs L , Lesavre P . Endothelium‐neutrophil interactions in ANCA‐associated diseases. J Am Soc Nephrol. 2012; 23: 1449–61. 22942199 10.1681/ASN.2012020119 PMC3431419

[42] Lauterbach M , O'Donnell P , Asano K , *et al* Role of TNF priming and adhesion molecules in neutrophil recruitment to intravascular immune complexes. J Leukoc Biol. 2008; 83: 1423–30. 18372339 10.1189/jlb.0607421

[43] Bretscher A , Edwards K , Fehon RG . ERM proteins and merlin: integrators at the cell cortex. Nat Rev Mol Cell Biol. 2002; 3: 586–99. 12154370 10.1038/nrm882

[44] Adada M , Canals D , Hannun YA , *et al* Sphingolipid regulation of ezrin, radixin, and moesin proteins family: implications for cell dynamics. Biochim Biophys Acta. 2014; 1841: 727–37. 23850862 10.1016/j.bbalip.2013.07.002 PMC3888837

[45] Adada MM , Canals D , Jeong N , *et al* Intracellular sphingosine kinase 2‐derived sphingosine‐1‐phosphate mediates epidermal growth factor‐induced ezrin‐radixin‐moesin phosphorylation and cancer cell invasion. FASEB J. 2015; 29: 4654–69. 26209696 10.1096/fj.15-274340 PMC4608912

[46] Adyshev DM , Moldobaeva NK , Elangovan VR , *et al* Differential involvement of ezrin/radixin/moesin proteins in sphingosine 1‐phosphate‐induced human pulmonary endothelial cell barrier enhancement. Cell Signal. 2011; 23: 2086–96. 21864676 10.1016/j.cellsig.2011.08.003 PMC3651873

[47] Nussbaum C , Bannenberg S , Keul P , *et al* Sphingosine‐1‐phosphate receptor 3 promotes leukocyte rolling by mobilizing endothelial P‐selectin. Nat Commun. 2015; 6: 6416. 25832730 10.1038/ncomms7416 PMC4396399

[48] Hall A . Rho GTPases and the actin cytoskeleton. Science. 1998; 279: 509–14. 9438836 10.1126/science.279.5350.509

[49] Singleton PA , Dudek SM , Ma SF , *et al* Transactivation of sphingosine 1‐phosphate receptors is essential for vascular barrier regulation. J Biol Chem. 2006; 281: 34381–93. 16963454 10.1074/jbc.M603680200

[50] Dudek SM , Jacobson JR , Chiang ET , *et al* Pulmonary endothelial cell barrier enhancement by sphingosine 1‐phosphate: roles for cortactin and myosin light chain kinase. J Biol Chem. 2004; 279: 24692–700. 15056655 10.1074/jbc.M313969200

