# Supplementary figures and images for: Sphingosine‐1‐phosphate (S1P) enhances glomerular endothelial cells activation mediated by anti‐myeloperoxidase antibody‐positive IgG
Source: J Cell Mol Med. 2017 Nov 23;22(3):1769–77. doi: 10.1111/jcmm.13458 (PMC5824416; doi:10.1111/jcmm.13458)

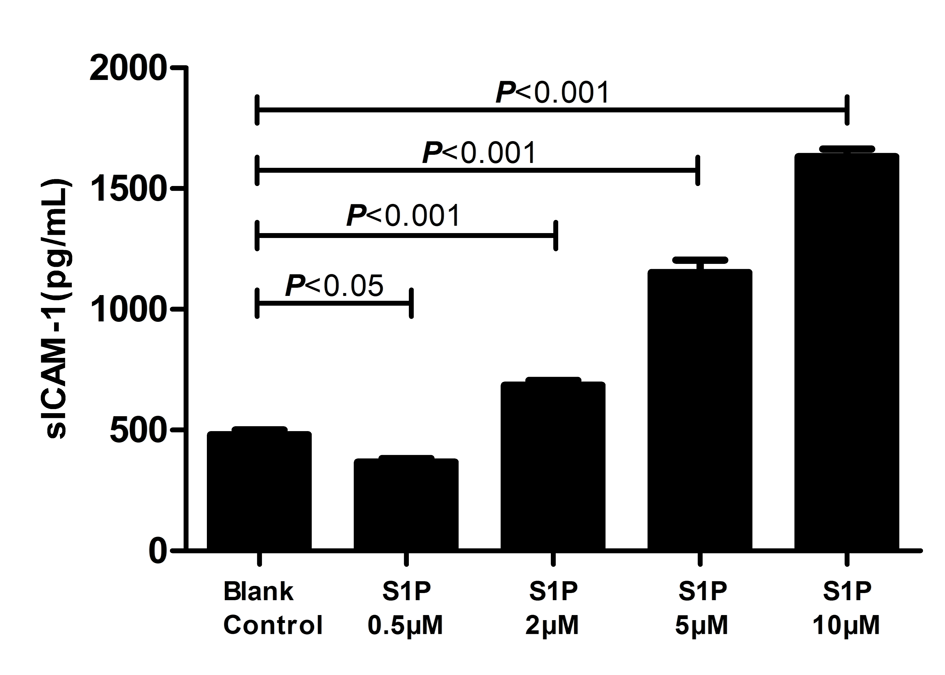

Supplement: Supplementary file 1 — Figure S1. Dose effect of S1P on sICAM‐1 expression in the supernatants of GEnCs. [file JCMM-22-1769-s001.tif]

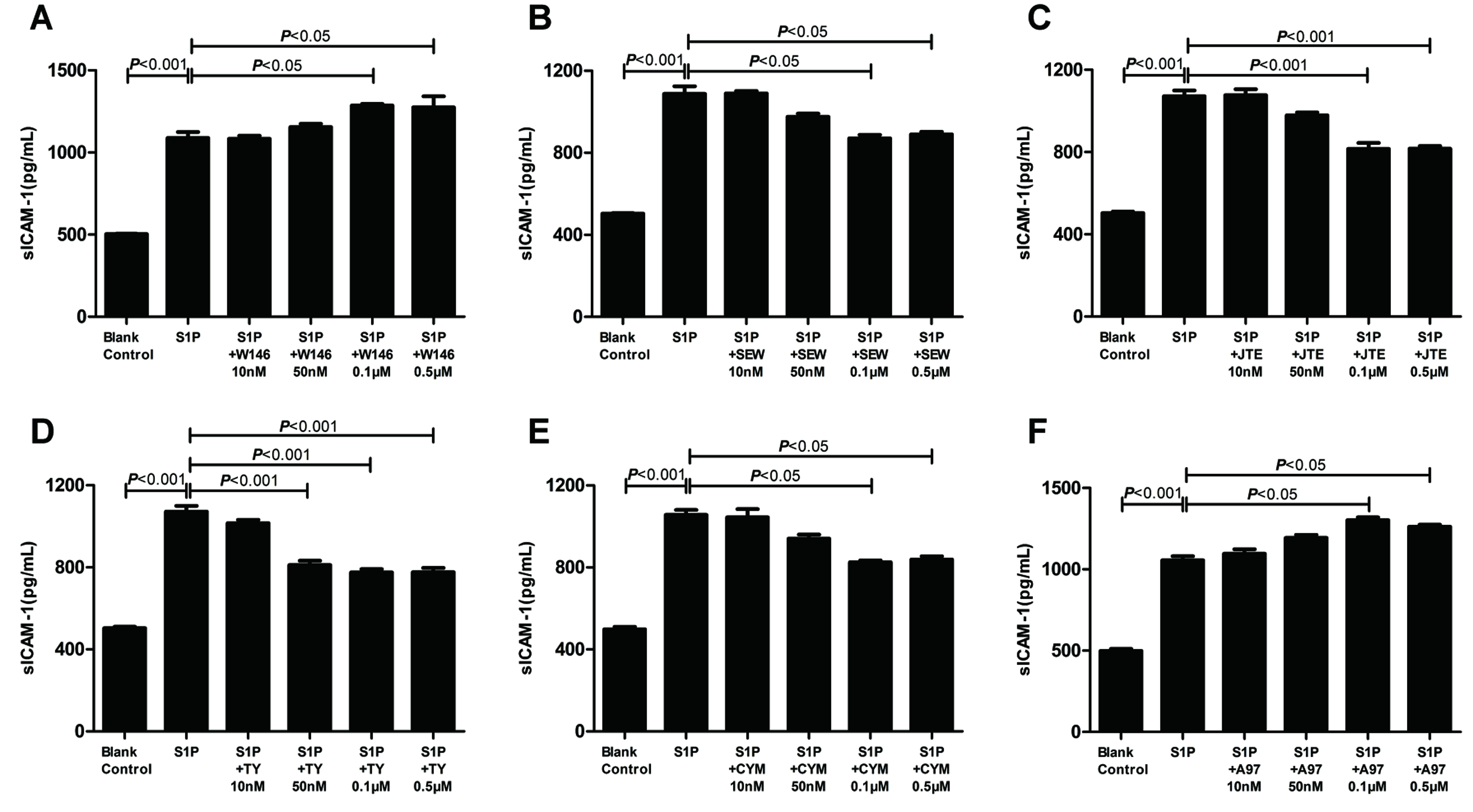

Supplement: Supplementary file 2 — Figure S2. Dose effect of S1PR agonists or antagonists on S1P‐induced sICAM‐1 expression. [file JCMM-22-1769-s002.tif]

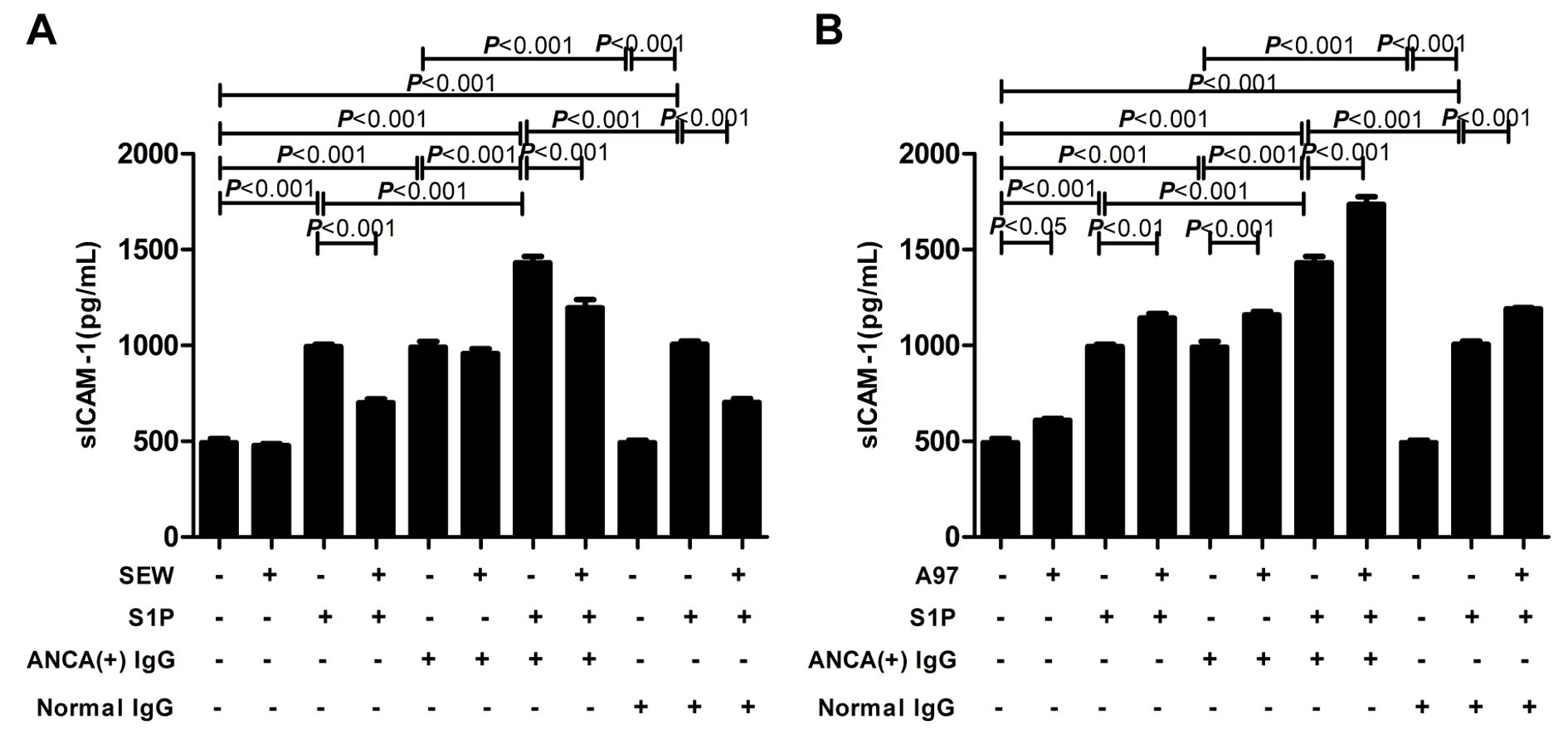

Supplement: Supplementary file 3 — Figure S3. Effect of S1PR agonists on sICAM‐1 level in the supernatants of GEnC stimulated by S1P plus MPO‐ANCA‐positive IgG. [file JCMM-22-1769-s003.tif]

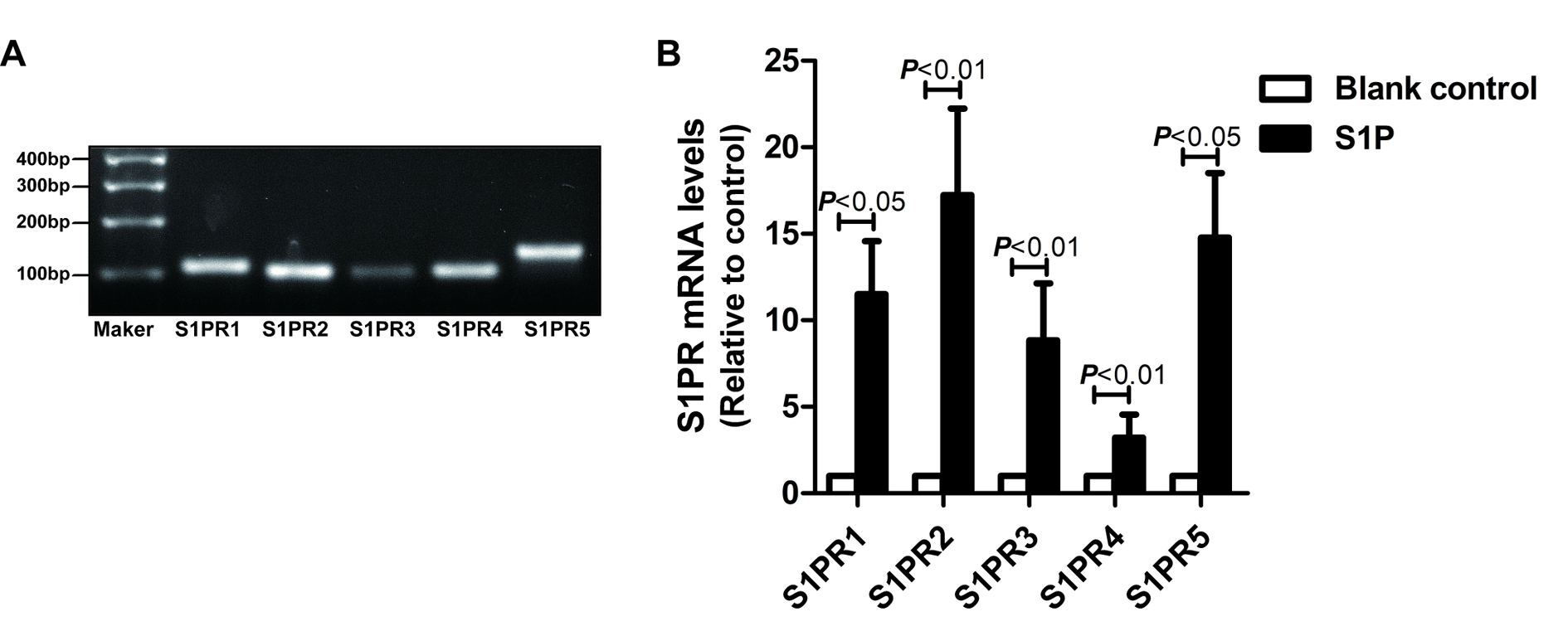

Supplement: Supplementary file 4 — Figure S4. Expression of S1PR1–5 in GEnCs were measured by RT‐PCR. [file JCMM-22-1769-s004.tif]
