# Supplementary material for: Sphingosine‐1‐phosphate (S1P) enhances glomerular endothelial cells activation mediated by anti‐myeloperoxidase antibody‐positive IgG
Source: J Cell Mol Med. 2017 Nov 23;22(3):1769–77. doi: 10.1111/jcmm.13458 (PMC5824416; doi:10.1111/jcmm.13458)
Supplement: Supplementary file 5 [file JCMM-22-1769-s005.doc]

**Supporting information**

**Figure legends**

**Figure S1. Dose effect of S1P on sICAM-1 expression in the supernatants of GEnCs**

**Figure S2. Dose effect of S1PR agonists or antagonists on S1P-induced sICAM-1 expression**

**Figure S3. Effect of S1PR agonists on sICAM-1 level in the supernatants of GEnC stimulated by S1P plus MPO-ANCA-positive IgG**

**Figure S4. Expression of S1PR1–5 in GEnCs were measured by RT-PCR**

A. the PCR products were size-fractionated in a 2% agarose gel

B. Expression of S1PR1-5 mRNAs were measured by real-time RT-PCR
